# Fatal Attraction of Short-Tailed Shearwaters to Artificial Lights

**DOI:** 10.1371/journal.pone.0110114

**Published:** 2014-10-15

**Authors:** Airam Rodríguez, Graeme Burgan, Peter Dann, Roz Jessop, Juan J. Negro, Andre Chiaradia

**Affiliations:** 1 Research Department, Phillip Island Nature Parks, Cowes, Victoria, Australia; 2 Department of Evolutionary Ecology, Estación Biológica de Doñana (CSIC), Seville, Spain; University of Veterinary Medicine Hanover, Germany

## Abstract

Light pollution is increasing around the world and altering natural nightscapes with potential ecological and evolutionary consequences. A severe ecological perturbation caused by artificial lights is mass mortalities of organisms, including seabird fledglings that are attracted to lights at night on their first flights to the sea. Here, we report on the number of fledging short-tailed shearwaters *Ardenna tenuirostris* found grounded in evening and morning rescue patrols conducted at Phillip Island, Australia, during a 15-year period (1999–2013). We assessed factors affecting numbers of grounded birds and mortality including date, moon phase, wind direction and speed, number of visitors and holiday periods. We also tested experimentally if birds were attracted to lights by turning the lights off on a section of the road. Of 8871 fledglings found, 39% were dead or dying. This mortality rate was 4–8 times higher than reported elsewhere for other shearwater species, probably because searching for fledglings was part of our systematic rescue effort rather than the opportunistic rescue used elsewhere. Thus, it suggests that light-induced mortality of seabirds is usually underestimated. We rescued more birds (dead and alive) in peak fledging, moonless and windy nights. Mortality increased through the fledging period, in the mornings and with increased traffic on holiday periods. Turning the road lights off decreased the number of grounded birds (dead and alive). While moon, wind and time are uncontrolled natural constraints, we demonstrated that reduction of light pollution and better traffic management can mitigate artificial light-induced mortality.

## Introduction

Natural parks aim to preserve natural conditions and encourage sustainable use by the public. Public visitation and engagement are essential for generating support and economic resources for nature conservation, but any detrimental effects that visitors have on biodiversity need to be managed (e.g. direct disturbance, road traffic, or habitat fragmentation; [1]). Light pollution is a frequently overlooked consequence of human visitation, mainly because a majority of visits to park and reserves occur during daylight hours. This pollution source can induce evolutionary and ecological disruptions on a wide range of biological processes, and thus, add to a plethora of threats challenging biodiversity conservation [2]–[4]. From a conservation perspective, one of the most severe perturbations caused by light pollution is episodes of mass mortality of organisms from different taxa, including birds, sea turtles and numerous insect species (e.g. [5]–[8]).

The conservation status of seabirds is deteriorating faster than any other group of birds [9] with petrels (Order Procellariiformes) occupying the top in the ranking [10]. Burrow-nesting petrel species (including shearwaters and storm-petrels) are nocturnally active at their breeding colonies, and their fledglings leave their nests at night, when they can be negatively affected by artificial lighting [11]. Worldwide, thousands of birds are attracted to lights every year during their first flights from their nests to the open ocean [12]–[17]; a phenomenon called ‘fallout’ [18]. Some fledglings may actually reach the ocean successfully but are attracted by the coastal lighting back onto the land [19]–[21]. Fledglings are vulnerable to injury or death by collisions with human infrastructure and once grounded, to predation or becoming road casualties. Through rescue campaigns conducted by the public, NGOs and local and regional governments, many fledglings are released back into the wild. However the basis for the attraction to or disorientation by fledglings requires more research [10]. Information coming from rescue campaigns can be crucial in shedding light on the extent of this ecological problem and its impact on these cryptic species (e.g. [22]–[25]).

**Figure 1:**
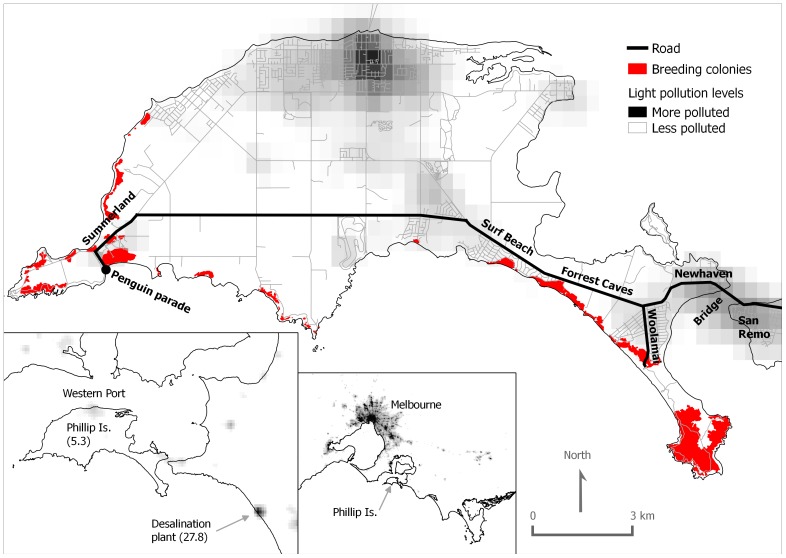
Phillip Island map showing the road where rescue patrols are conducted, light pollution levels and the main Short-tailed shearwater (*Ardenna tenuirostris*) breeding colonies. Light pollution levels are taken from a cloud-free composite of VIIRS night time lights corresponding to April and October of 2012 and produced by the Earth Observation Group, NOAA National Geophysical Data Center (available at http://ngdc.noaa.gov/eog/viirs/download_monthly.html). The maximum light pollution levels for Phillip Island and Wonthaggi desalination plant are in brackets (nW/sr*cm^2^).

We used information gathered during short-tailed shearwater *Ardenna tenuirostris* rescue campaigns conducted at Phillip Island, Victoria, Australia, from 1999 to 2013 to report the number of affected birds and the temporal and spatial patterns of groundings. Phillip Island differs from other locations where fallout has been studied (e.g. Azores, Canary Islands, Hawaii or La Reunion Island), in the larger size of the shearwater breeding population and the lower altitude and lower light pollution levels. Phillip Island is an “Important Bird Area” supporting more than 1% of the global populations of both short-tailed shearwaters and little penguins *Eudyptula minor* [26]. Ecotourism is a major economic activity on the island. Visitors can watch little penguins crossing the beach at evening at the Penguin Parade (Phillip Island Nature Parks, PINP, www.penguins.org). The activity attracts over half a million visitors each year, providing crucial financial support to the conservation and management of island wildlife through ecotourism [27]. However, it also increases road traffic at certain hours (approx. 2 hours before and after sunset) and consequently also light pollution coming from road and vehicle lights [28]. Visitors, mainly coming by road from Melbourne, have to cross a bridge to get onto the island and drive to the Penguin Parade (Figure 1).

We have assessed potential factors determining the number and the light-induced mortality of grounded birds by artificial lights per night. Short-tailed shearwater fledglings seem to need strong winds to take off on their initial flights [29], although no detailed studies have been conducted on this issue in petrels [30]. Therefore we anticipated wind speed could have a positive relationship with the number of birds grounded and the proportion of dead birds (mortality). Given the location of breeding colonies in relation to the roads where rescue patrolling is conducted (Figure 1), we expected that fledglings would drift towards artificial lights with southerly or westerly winds. We also tested the potential effect of the holiday periods or visitor numbers, expecting higher number of birds grounded during nights with higher numbers of vehicles. We included moon phases and date in the analyses as a majority of shearwaters are grounded during moonless nights and around the peak fledging period elsewhere [12], [14]–[16]. Finally, the relative calm water of the adjoining bay, Western Port, may be an important area for fledglings as they can rest and preen before starting migration to North Pacific Ocean. The bridge to the island may constitute a barrier for fledglings coming into or out of the bay. Aiming to mitigate mortality, PINP in collaboration with VicRoads (State Government department with responsibility for roads in Victoria) and SP AusNet (VicRoads lighting contractor) turned lights off on the bridge to the island during some days of the fledging period. We evaluate whether this lighting management had any effect on the attraction of fledglings to lights.

## Material and Methods

### Ethics statement

Birds were picked up and rescued under the provisions of the Victorian State Government's Prevention of Cruelty to Animals Act (1986), Australia. No ethics permit was required as this was not a scientific experiment; it was a management action.

### Study area and shearwater rescue patrols

Phillip Island is located in south-eastern Australia (38°29′; 145°15′). It is 26 km long and 9 km wide, with an area of about 100 km^2^. Relief is mostly flat, and its maximum altitude is about 112 m above sea level. It holds an important short-tailed shearwater breeding population which is mainly distributed around the south coast (Figure 1). Artificial lights are visible from some colonies, e.g. from the largest one located at Cape Woolamai, while other colonies show unpolluted skies at ground level, but some glow or lights are visible once the birds are in the air. The resident human population is about 10,000 people, but it increases dramatically during summer holidays or special events. The island is connected to the mainland by a 640 m long bridge, which is illuminated for road safety reasons by low pressure sodium vapour lights fitted in aeroscreen lamps. The bridge has concrete walls approximately 0.6 m high along the road without any exit points for grounded animals. Therefore, birds grounded on the bridge have a high chance of being run over by vehicles, because they rarely take off until they have rested which may take some hours and after dawn they will not take off at all (G.B. pers. obs.).

The shearwater rescue patrols started in 1999 as part of a public awareness program to reduce the road mortality of shearwater fledglings attracted to road lights and to educate the community of the cultural and natural history of the shearwaters. Patrols were conducted twice at night (between 17:00–22:30 h local time) and morning before sunrise (between 5:30–7:00 h) from 18 April to 8 May, coinciding with the fledging season of the short-tailed shearwaters. During rescue patrols, at least two PINP rangers drove in a vehicle along the main roads searching for grounded shearwaters (Figure 1). Rescued birds were examined for injuries before being released in a breeding colony. Injured birds were admitted to the PINP wildlife rehabilitation clinic. Numbers of individuals for each night were only available for the 2007–2013 period when date, time of the day (night or morning), status (alive or dead) and location were recorded. Data from 1999 to 2006 were recorded per year (status and location) and used for assessing temporal patterns of the numbers and mortality of grounded birds. To study spatial distribution of the fallout, roads were divided into seven main areas: Summerland, Surf Beach, Forrest Caves, Cape Woolamai, Newhaven, Bridge and San Remo (Figure 1). Because night traffic on Summerland is concentrated in a few hours after dusk when visitors are leaving the Penguin Parade, an increased rescue effort was conducted there at these times. This has involved stopping traffic to get birds off the road. The lights on the bridge were turned off during some days of the fledgling season in an attempt to learn more about their effects on the fallout and whether it would reduce fledgling mortality on the bridge.

### Impact on the population

To roughly estimate the percentage of fledglings affected by light attraction (grounded birds divided by estimated number of fledglings produced by the population) and to compare with other studies, we followed procedures of Le Corre and co-workers [14]. The number of fledglings produced per year was calculated multiplying population size (542 300 nests; [31]) by breeding success (0.41 and 0.68 eggs/chicks -min and max values; [29]) and proportion of breeders (0.79 and 0.86 nests with eggs -min and max values; [29]). These average breeding rates are in the range of average rates for most petrels [30]. We assumed that this estimated breeding population did not change during the study and we used the highest number of grounded birds in a year (1233 birds in 2013) as a worst-case scenario. Our estimated rates are conservative and should be interpreted as minimal numbers because of error in detecting birds, i.e. some birds could never be rescued, or being grounded in areas where no rescue patrols is conducted.

### Explanatory variables

To model the number of grounded birds, fledging peak date was standardised as 0 for the 27 April (median date for the total number of grounded birds; Figure S1). Thus, negative and positive values were assigned to anterior and posterior fledging peak dates respectively (e.g. 25 April  = −2; 5 May  = +8). To model the proportion of dead birds, date was used as a simple term because a linear relationship was expected [32]. The fraction of moon disc illuminated per night was used as a quantitative way to describe the lunar phase, where 0 and 1 indicate new and full moon, respectively. It was obtained from the U.S. Naval Observatory website (http://aa.usno.navy.mil/data/docs/MoonFraction.php). Wind data (speed in km/h and direction in degrees) were obtained from an automated meteorological station located at Rhyll, Phillip Island, with an elevation of 13 m (station reference: 086373, the National Climate Centre, Bureau of Meteorology, Australia). Because wind information was collected every three hours in a day, we assigned the values recorded at 06:00 and 21:00 hours to morning and night patrols, respectively. Wind direction in degrees (0° corresponds to North, and 180° corresponds to South) was converted into radians (θ; 0 and 2π radians correspond to North, and π radians corresponds to South). Cos(θ) and sin(θ) transformations were included in the statistical models as explanatory variables to investigate possible effects of wind direction on the number of shearwater fledglings grounded on the roads. The public holiday variable was created as a two-level factor. Days were classified as ‘holiday’, if they were Saturday, Sunday, ANZAC holiday (25 April) or Easter holidays, or as ‘working day’ for the remaining days. Number of visitors to the Penguin Parade per night was provided by the ticketing system of the PINP. Finally, we created a two-level factor (bridge lighting) to describe when bridge lights were turned on or off in 2009, 2012 and 2013. Lights were turned off from 24 to 28 April in 2009, from 26 April to 1 May in 2012 and from 23 April to 1 May in 2013.

### Statistical analysis

We used Generalized Linear Models (GLM) to model the effect of date, moon, patrol, holiday period and direction and speed of wind on the number of grounded birds. Negative binomial error distribution and log link were used because of the high overdispersion of count data (residual deviance: 3849.9 on 278 degrees of freedom for the full model with Poisson error distribution).

To model the proportions of dead birds per night or per year we used GLMs with a two-column object for response (dead/alive birds), quasi-binomial error distribution and logit link [33]. Date, moon, patrol, holiday period and wind speed were included as explanatory variables. For mortality models, we selected a subset of data, excluding Summerland and Woolamai sections of road. Because of the higher effort conducted on the Summerland and the low traffic intensity on the Woolamai sections, mortality rates at these places were low, masking potential effects of holiday periods or number of visitors. Results for whole dataset are supplied in the Supporting Information.

To test potential linear trends in the proportion of dead birds throughout the years, a second GLM was run including year as a continuous explanatory variable and using data from the whole study period (1999–2013).

To model the effect of number of visitors to the Penguin Parade on the number of grounded birds and the proportion of dead birds, we sub-selected data for the night patrol (when visitors leave the Penguin Parade). We run models only including the significant explanatory terms from the averaged models and the number of visitors.

To model the effect of the light management at the bridge on the number of grounded birds, we used GLMs with error distributions and link functions as above. To avoid over parameterization due to the smaller dataset (note that we used data from 2009, 2012 and 2013, i.e. when lights were turned off), we only included peak fledging date, (peak fledging date)^2^, rescue and lighting management as covariates.

Collinearity was tested in the two global models (number of birds and mortality models), but it was not an issue (largest Variance Inflation Factor  = 1.237). Multi-model inference was used to identify the best possible models based on information criteria (the lowest information criteria, the best model) and to rank all independent variables according to their influence on the response [34]. QAICc and AICc were used as information criteria for quasi-binomial and negative binomial distribution models, respectively. The best fit models in the final selection, i.e. models within two AICc (or QAICc) units from the best model, and their Akaike weight of evidence (*w*) were used to estimate averaged regression coefficients. *w*_i_ were calculated within the set of best models (delta <2). Explanatory variables were ranked by importance, i.e. sum of their *w* over all competing models (the closest to 1, the highest importance), and significance was reached when 95% confidence intervals (CI) did not include zero. We used the glm and glm.nb functions, as well the MASS, car and MuMIn packages in R version 3.0.3 (R Foundation for Statistical Computing, Vienna, Austria).

## Results

### Numbers, temporal and spatial patterns

**Figure 2:**
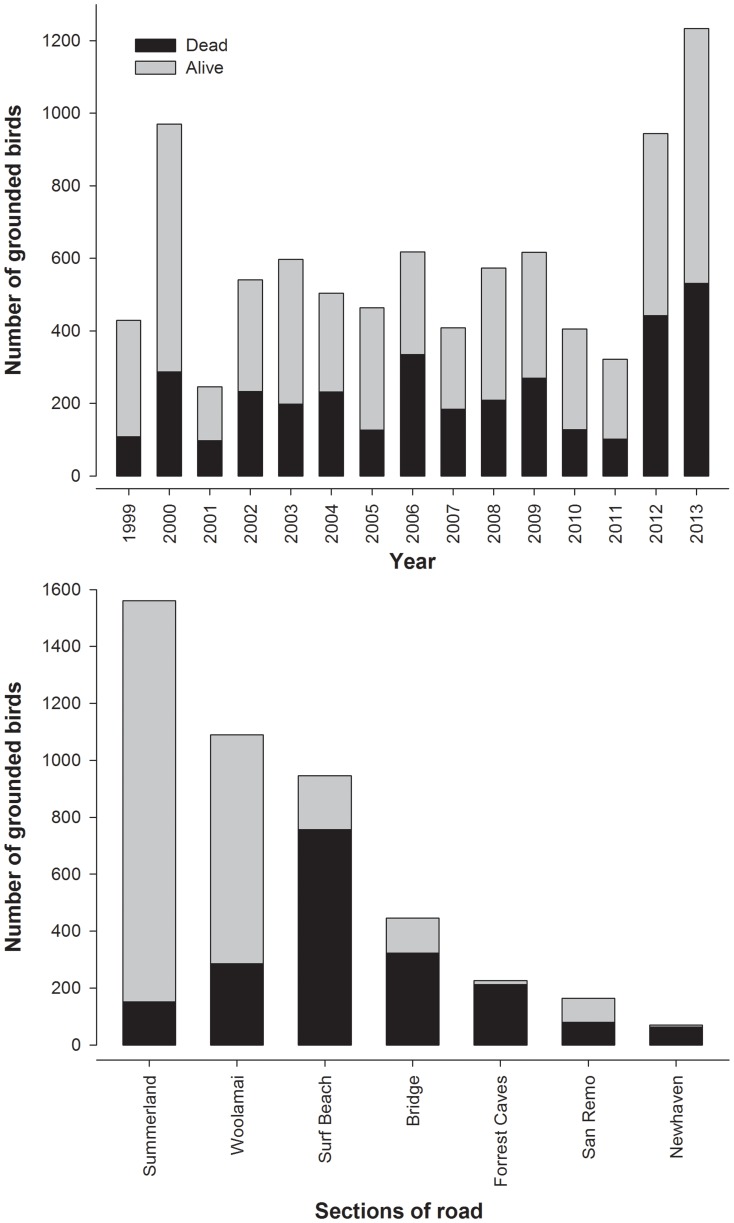
Numbers of grounded birds on Phillip Island per year and section of road during 1999–2013.

A total of 8871 birds were collected during the 1999–2013 rescue campaigns. A large variation exists in the number of birds affected per year (mean ±SD = 591.4±266.5 birds; range  = 246–1233; Figure 2). No temporal trend was observed (Spearman Rank Correlation  = 0.19, *P* = 0.490). The highest numbers of grounded birds were found in those areas close to the breeding colonies (Figures 1 & 2). We estimated that less than 1% of the fledglings produced annually are affected by attraction to artificial lights (depending on min. and max. breeding success and breeder proportion values, our estimates range between 0.39 and 0.70%).

### Factors determining the number of grounded birds

**Figure 3:**
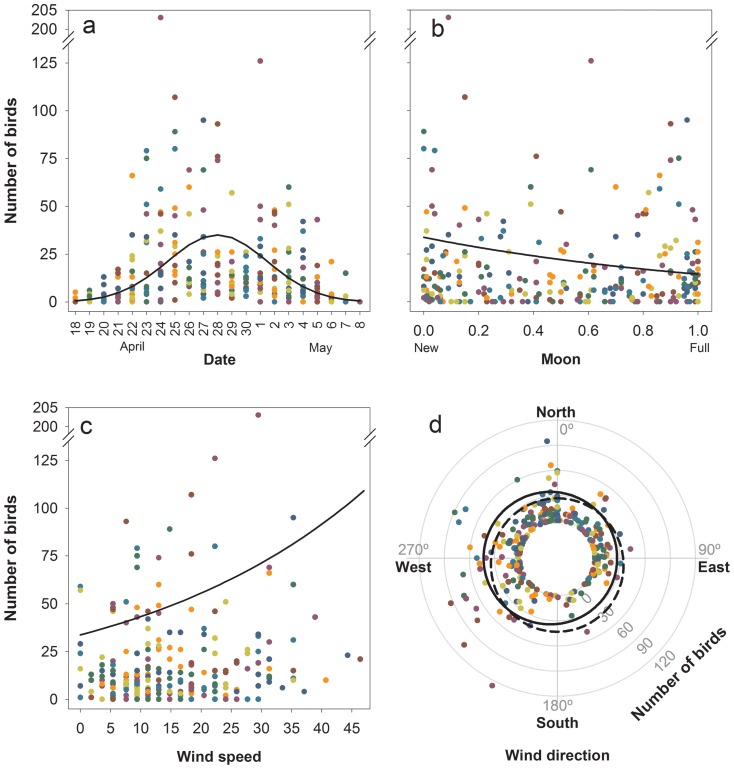
Effects of the date (a), moon (b), wind speed (c) and wind direction (d) on the numbers of birds grounded according to the model averaging. Dots indicate the rough data and the lines the fitted model. Solid and dashed lines represent sin(wind direction) and cos(wind direction) respectively (d). Radial axe scale has been modified to improve visualization (d).

Multi-model inference selected three models (Table S1 in File S1). At least seven explanatory variables reached importance values higher than 0.85 and five of these variables were included in all competing models (Table 1). According to the average estimates, date had an important quadratic effect on the number of grounded birds, reaching maximum numbers during the middle fledging season (around 27–28 April; Figure 3a). More birds were grounded during the night patrols than during the morning ones. The number of grounded birds was negatively correlated with the fraction of moon disc illuminated by the sun and positively with the wind speed (Figures 3b & 3c). Variables quantifying the effect of wind direction on the number of grounded birds obtained high importance values (0.77 and 0.75, Table 1). The negative sign of the estimates indicate that higher numbers of birds were grounded when wind blows from west or south (Figure 3d). Holidays had no effect on the number of birds grounded (Table 1). Number of visitors to the Penguin Parade was not related to the number of grounded birds (95% CI: [−0.0005, 0.0002]), even when modelling a sub-selection of data from Summerland.

**Table 1:** Results of the model averaging for the best Generalised Linear Models explaining the number of grounded birds, the proportion of dead birds and the effect of lighting management on the number of grounded birds at the Phillip Island bridge in 2009, 2012 and 2013.

Response var.	Explanatory var.	Importance	Averaged estimate	S.E.	95% confidence intervals	
Number of birds						
	Intercept		3.517	0.207	3.110	3.924
	**Date**	1	0.081	0.015	0.052	0.110
	**(Date)^2^**	1	−0.041	0.003	−0.046	−0.036
	**Moon**	1	−0.848	0.211	−1.263	−0.433
	**Rescue Patrol** *	1	0.384	0.145	0.098	0.669
	**Wind Speed**	1	0.025	0.008	0.009	0.040
	cos(Wind Direction)	0.77	−0.243	0.118	−0.474	−0.011
	sin(Wind Direction)	0.75	−0.220	0.111	−0.438	−0.002
	Holidays*	0				
Proportion of dead birds						
	Intercept		1.048	0.452	0.156	1.941
	**Date**	1	0.052	0.016	0.021	0.083
	**Rescue Patrol** *	1	−1.540	0.123	−1.783	−1.296
	**Holidays** *	1	−0.772	0.140	−1.048	−0.497
	Moon	0.40	0.280	0.176	−0.067	0.627
	Wind Speed	0				
Number of birds (bridge)						
	Intercept		1.929	0.343	1.250	2.608
	**(Date)^2^**	1	−0.055	0.010	−0.074	−0.036
	**Rescue Patrol** *	1	−1.807	0.365	−2.530	−1.084
	**Lighting Management** *	1	1.273	0.459	0.364	2.183
	Date	0.62	0.074	0.038	−0.002	0.149

*‘Morning’ rescue patrol, ‘holiday’ and ‘light off’ levels are taken as reference.

Variables in bold are those included in all competing models (see File S1 for multi-model inference details).

### Mortality

Thirty nine percent of birds (3481 out of the 8871 total birds found grounded) were found dead or dying. The proportion of dead birds did not increased during the years (Estimate ±SE = 0.035±0.018; 95% CI = [−0.0007, 0.0703]). The locations with the highest mortality in numbers were Surf Beach (756 dead birds) and on the bridge (323 dead birds), while the percentage of mortality was the highest along the Forrest Caves and Newhaven sections (94% and 91% of the birds found at these locations died, respectively).

**Figure 4:**
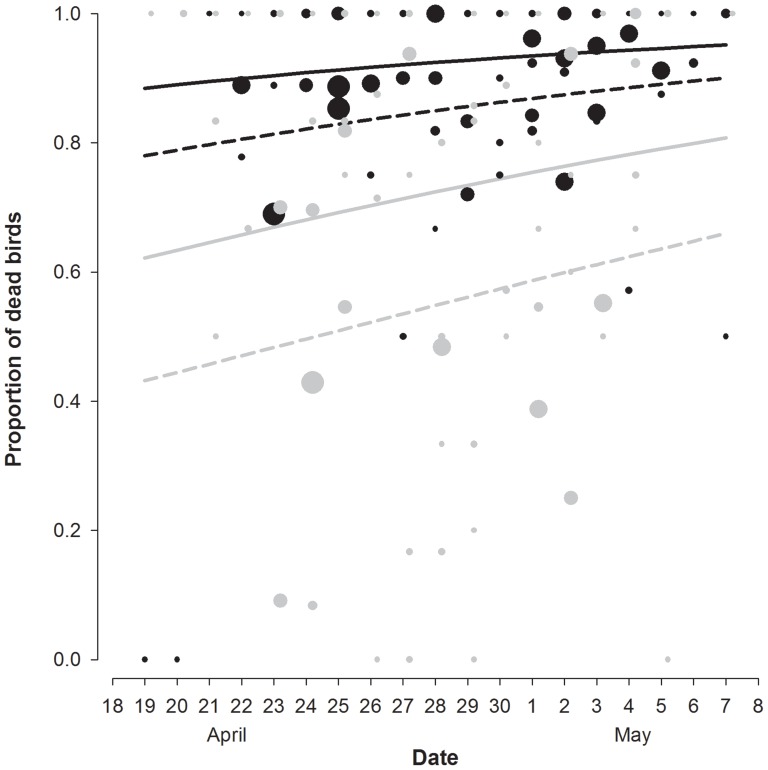
Effect of date on the proportion of dead birds grounded. Dots indicate the rough data and the lines the fitted model. Rescue patrolling is distinguished by colour (Grey: Night, Black: Morning) and holiday periods by line type (Dashed: working day; Solid: holidays).

Multi-model inference selected two models (Table S2 in File S1). Three explanatory variables were included in all competing models (Table 1). The proportion of dead birds was positively related to date. More dead birds were found during the morning than during the night patrols and during holiday than during working days (Figure 4). Wind speed and the fraction of moon disk did not contribute to explaining variation in patterns of mortality (Table 1). Results for the whole dataset, i.e. including all locations (see Statistical Analyses section) showed the same results, except that holiday was not an important variable (Tables S3 & S4 in File S1). Number of visitors to the Penguin Parade was not related to mortality (95% CI: [−0.0005, 0.0007]).

### Lighting management

The GLM explaining the number of grounded birds at the bridge for the years 2009, 2012 and 2013, and including peak fledging date, (peak fledging date)^2^, rescue and lighting management as explanatory variables (global model), obtained the lowest AICc. The following candidate model (delta AICc = 0.97) dropped off peak fledging date. The remaining models obtained AICc values higher than two units from the best model (Table S5 in File S1). Lighting management was included in the two best fit models and it had a significant effect on the number of grounded birds at the bridge, decreasing the numbers when lights were turned off (Table 1).

## Discussion

### Numbers of birds

The maximum number of grounded short-tailed shearwaters per year at Phillip Island is similar to other species on other islands, but we have to note the huge differences in breeding population size between islands. The percentage of fledglings affected by lights on Phillip Island is low in comparison to other studies on different species (Table 2). Apart from species-specific attraction to lights, two main non-exclusive factors could explain these differences: 1) the low light pollution levels of Phillip Island in relation to other more populated islands like Tenerife (Canary Islands, Spain), La Reunion (Indian Ocean, France) or Sao Miguel (Azores, Portugal) (Table 2); and 2) the spatial distribution of breeding colonies in relation to lights. It has been suggested that species breeding on inlands, having to cross over cities, are likely to be more vulnerable to light pollution [14], [24]. Given the proximity of breeding colonies to the sea at Phillip Island (Figure 1), many fledglings can avoid flying over or near lit areas at night before reaching the ocean. However, the very flat landscape of Phillip Island may allow birds to see lit areas from fledgling routes to the ocean [20]. In fact, it has been proposed that fledglings could be attracted to lights once they have reached the sea [19], [21]. At this point, it is worth to mentioning information coming from the recently built Wonthaggi Desalination Plant. This factory is located on mainland Australia about 15 km from the closest shearwater breeding colony (Cape Woolamai) and produced light pollution levels around 27 nW/sr*cm^2^ during the construction phase, which is 5 times higher than the most light-polluted area on Phillip Island (Figure 1). In 2012, 237 fledglings were grounded at this site, which constituted the equivalent to one-quarter of all grounded birds on Phillip Island in the same year and supports the hypothesis that fledglings can be attracted to lights after reaching the ocean.

**Table 2:** Comparison of the rescue campaigns worldwide.

Island	Species	Maximum numbers (year)	Affected fledglings (%)	Mortality (%)	Maximum pollution level (nW/sr*cm^2^)	Altitude (m above sea level)	Source
Tenerife, Canary Islands	*Calonectris diomedea*	1765 (2010)	45.4–60.5	4.8	103.6	3718	[15], [32]
	*Bulweria bulwerii*	43 (1995)	6.4–8.6	11.8	103.6		[15], [25]
	*Puffinus baroli*	44 (1995)	20.9–46.9	4.9	103.6		[15], [25]
La Reunión Island	*Pterodroma baraui*	∼830^a^ (2001)	20–40	10	68.7	3071	[14], [39]
	*Puffinus balloni*	283 (1999)	10–17	8	68.7		[14]
Sao Miguel, Azores	*Calonectris diomedea*	526 (2009)	16.7	14	52.1	1103	[37]
Faial, Azores	*Calonectris diomedea*	1236 (2008)	19.7	4	28.8	1043	[17]
Kauai, Hawaii	*Puffinus newelli*	2220 (1987)	15^b^	9	16.5	1598	[13], [35]
Pico, Azores	*Calonectris diomedea*	1547 (2008)	15.2	8	15.0	2351	[17]
Phillip Island, Australia	*Ardenna tenuirostris*	1233 (2013)	0.39–0.70	40	5.3	112	This study

a Estimated from Figure 1 in [39];

b Estimated by [13].

Table shows the maximum numbers of grounded fledglings in one year (the year is in brackets), the percentage of affected birds following [14], the percentage of dead birds rescued, the maximum light pollution level on the island according to a cloud-free composite of VIIRS nighttime lights corresponding to April and October of 2012 (Earth Observation Group, NOAA National Geophysical Data Center; available at http://ngdc.noaa.gov/eog/viirs/download_monthly.html) and the maximum altitude. It is not an exhaustive list.

Trends in numbers of fledglings grounded by lights have provided useful information to estimate population trends on cryptic petrel species breeding in inaccessible and remote areas [13], [25], [35]. The short-tailed shearwater global population was estimated at 23 million individuals in 1985 [29] and the global population trend appears to be decreasing [10], [26]. Annual breeding success could be related to the number of grounded birds, but it has not been recorded during the study period precluding an assessment of its effect.

### Factors determining the number of grounded birds

Higher numbers of grounded fledglings are expected during adverse weather conditions, i.e. on overcast and rainy nights [12], probably because of the potential of clouds, mist and rain to increase light pollution levels [36]. However, the potential effect of wind has not been proposed as a factor determining the number of grounded birds previously. In this study, after controlling for the curvilinear effect of peak fledging date and the influence of the moon phase, we found that nights with the highest speed winds resulted in the largest numbers of grounded birds. Shearwaters need a long runway to take off, even more critical for fledglings that are not flight-experienced [29]. Thus, wind speed could be more important for determining fledging on flat islands like Phillip Island, because birds cannot take advantage of mountain winds or even jump from high cliffs or deep slopes [30]. This could explain why wind speed has not been previously identified as an influential factor of the number of grounded birds on other islands with higher elevations (Table 2).

The location of the breeding colonies in relation to areas with artificial lights on Phillip Island may have also enhanced the effect of wind direction on the numbers of grounded birds. The lit areas at night are on the lee side of prevailing strong winds blowing from south or west, when the highest numbers of grounded birds were recorded. Thus, birds are probably blown away from the colony onto the roads. In the majority of other studies of breeding colonies on islands, the colonies are located all around the coast and, in some cases, inland (see [14], [20], [25], [37]), while light-polluted areas are usually located around the coastal cities. In this context, the effect of wind direction on the number of grounded birds is hard to test.

### Mortality

Despite a low proportion of affected fledglings in relation to the overall production of the population, our study indicates than at least 39% of fledglings affected by light attraction die. This rate is 4–8 times higher than those reported in other rescue campaigns involving other species and islands, suggesting that light-induced mortality may be considerably underestimated elsewhere (Table 2). These other rescue campaigns were opportunistic, normally involving the collaboration of the general public. While members of the public may be more inclined to collect live birds than dead ones, PINP rangers actively searched for dead or live fledglings. In this sense, a similar mortality rate (43%) was estimated when an active search for birds was conducted on Kauai, Hawaii [13], [19].

On Phillip Island, the main cause of mortality of short-tailed shearwater fledglings once they have been grounded by light pollution is collisions with vehicles, although introduced predators and domestic pets may kill some birds. It explains why morning rescue patrols found a higher number of dead birds than night rescue patrols. During night rescue patrols, the time spent by fledglings on the road is shorter as they do not leave their nests before sunset, while during the morning, fledglings could have spent much of the night on the road increasing the chances of being run over by vehicles or predated.

The number of grounded birds was not related to holiday periods or the number of visitors to the Penguin Parade. This suggests that light pollution levels did not significantly increase as a consequence of the higher occupation of holiday houses or from increased vehicle lights during the Penguin Parade hours or even cars themselves. However, mortality increased during holidays probably due to increased traffic. Thus, two threats interact in a fatal combination: light pollution disorients birds until they get stranded and later traffic kills them. When mortality was model averaged with the whole dataset (including all sites), holiday did not reach significance (Tables S3 & S4 in File S1), confirming that the higher effort conducted by the rescue patrolling at Summerland reduced mortality. At Summerland, traffic speed limit was reduced from 80 to 40 Km/h, traffic signals displayed ‘drive safely, birds on road’ and road traffic was occasionally halted by PINP rangers to rescue grounded birds on road. In addition, public was alerted at the Penguin Parade (before leaving) to watch out for birds on the road.

Short-tailed shearwater mortality increased through the fledging season, similar to Cory's Shearwaters *Calonectris diomedea* on Tenerife [32]. Because mortality was also positively related to down cover density on the Canaries, Rodríguez and co-workers suggested fledglings were probably forced to fledge at the end of fledging period, when they were still not fully developed and not yet ready for fledging [32]. Another possibility could be that the fledglings are more exhausted at the end of the fledging period because of the effects of the desertion period [29] and their abilities to fly and to avoid road traffic collisions are more limited. More research is needed to clarify this point.

### Turning off lights

Despite the fatal attraction of petrels to lights being known for decades (see Imber 1975 and references therein), few experimental studies have tested its causes or explored mitigation actions. Shielding upward light emission decreased the number of Newell's shearwater *Puffinus newelli* fledgling groundings by nearly 40% [18]. In addition, light emission reductions (by turning lights off) also decreased the numbers of Leach's storm-petrels *Oceanodroma leucorhoa* [16]. Our study adds evidence to these two cases. Lighting management on the bridge decreased the number of grounded birds, reducing the chance of fatalities as birds grounding on the bridge are highly susceptible to be run over by vehicles.

The joint collaboration of community, PINP and State government road management (VicRoads) towards turning off street lights on the bridge on selected nights is a milestone in the management of a conflict of interests involving road security, light pollution and wildlife. Still, further simple improvements could be implemented to mitigate this mortality. First, the light turning off period should be extended to cover the whole fledging period. Second, a greater effort should be put into the morning rescue patrols, as a larger number of birds are found at this time of the day. Finally, the possibility of halting traffic on certain roads at critical times should be evaluated so that rescue patrol staff may search and rescue grounded birds with improved safety.

### Conclusions

Evaluation of population size and trends are first priorities in conservation research topics for seabirds [10]. That includes the understanding of all sources of mortality, natural or artificial, so that human-induced sources can be mitigated. Light pollution and its fatal attraction to seabirds is a growing problem worldwide as light pollution is increasing at a rate of 6% per year [38]. Although a low, and probably underestimated, rate of groundings is commonly recorded, we have shown here that mortality of shearwaters caused by lighting can be mitigated by turning lights off and regulating traffic (speed limit reduction, traffic stopping and display of warning signals).

## Supporting Information

Figure S1 **Mean and standard deviation (dots and whiskers, respectively) of the number of grounded birds per day during the period 2007–2013.** For simplification, whiskers have only been represented above the mean.
(JPG)

File S1 **Details of the best fit models explaining the number of grounded birds, the proportion of dead birds and the effect of lighting management on the number of recued birds at the bridge of Phillip Island.**
(PDF)
