# Supplementary figures and images for: Fatal Attraction of Short-Tailed Shearwaters to Artificial Lights
Source: PLoS One. 2014 Oct 15;9(10):e110114. doi: 10.1371/journal.pone.0110114 (PMC4198200; doi:10.1371/journal.pone.0110114)

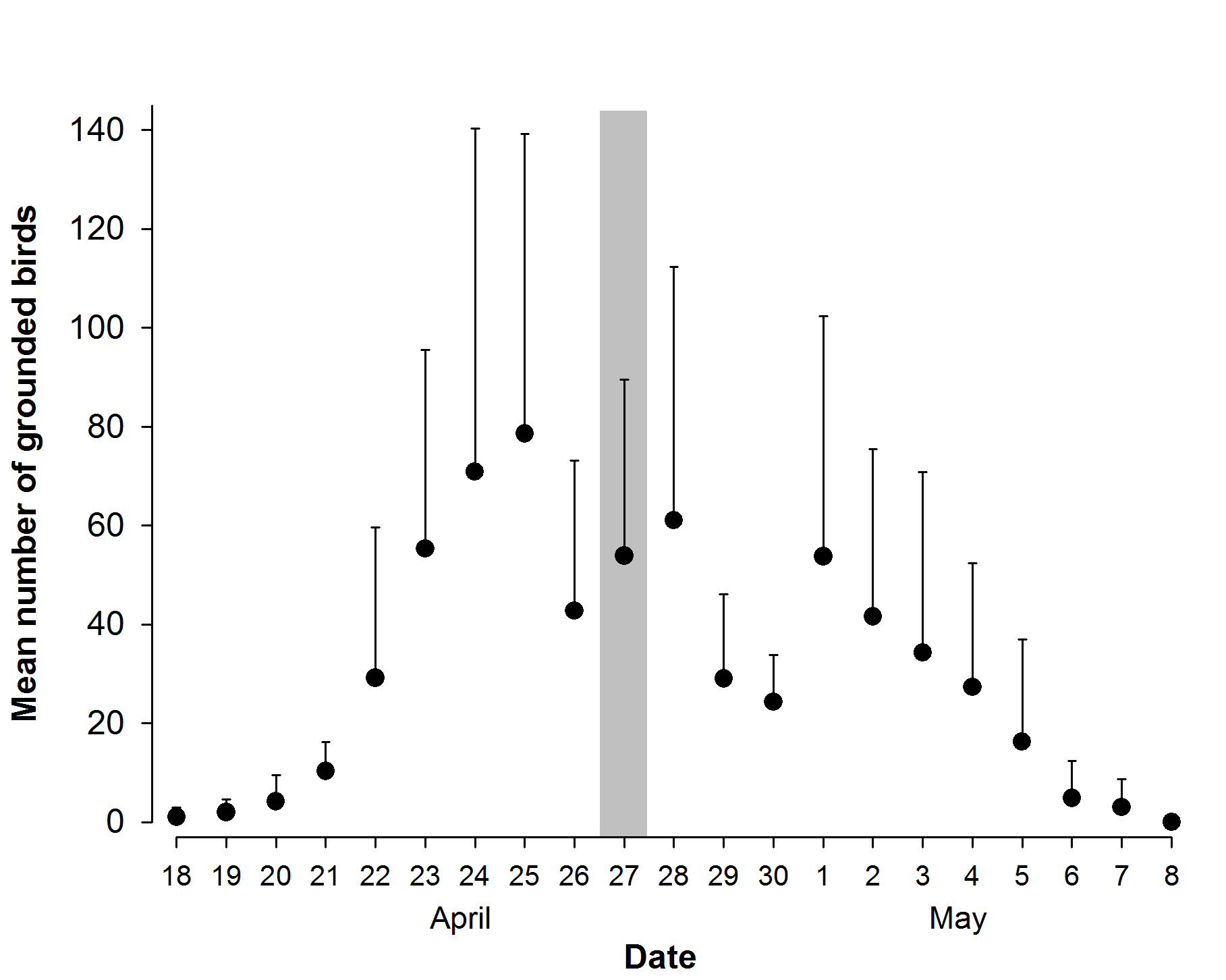

Supplement: Figure S1 — Mean and standard deviation (dots and whiskers, respectively) of the number of grounded birds per day during the period 2007–2013. For simplification, whiskers have only been represented above the mean. (JPG) [file pone.0110114.s001.jpg]
