# Supplementary material for: Fatal Attraction of Short-Tailed Shearwaters to Artificial Lights
Source: PLoS One. 2014 Oct 15;9(10):e110114. doi: 10.1371/journal.pone.0110114 (PMC4198200; doi:10.1371/journal.pone.0110114)
Supplement: File S1 — Details of the best fit models explaining the number of grounded birds, the proportion of dead birds and the effect of lighting management on the number of recued birds at the bridge of Phillip Island. (PDF) [file pone.0110114.s002.pdf]

Supporting Information

File S1. Details of the best fit models explaining the number of grounded birds, the proportion of dead birds and the effect of lighting management on the number of recued birds at the bridge of Phillip Island.

Table S1. Details of the best fit models explaining the number of grounded birds. Variable numbers: 1 = Date, 2 = $(\text{Date})^2$, 3 = Cos(Wind Direction), 4 = Moon, 5 = Rescue Patrol, 6 = Sin(Wind Direction), 7 = Wind Speed, 8 = Holidays (note that it is not included in the best fit models)

Variables	df	logLik	AICc	Delta	Weight
1234567	9	-941.08	1900.82	0.00	0.53
123457	8	-942.91	1902.34	1.53	0.25
124567	8	-943.00	1902.51	1.70	0.23

Table S2. Details of the best fit models explaining the proportion of dead birds. A sub-selection of the whole dataset, excluding Summerland and Woolomai areas, was used. Variable numbers: 1 = Date, 2 = Moon, 3 = Rescue Patrol, 4 = Holidays, 5 = Wind Speed (note that it is not included in the best fit models)

Variables	df	logLik	AICc	Delta	Weight
134	4	-292.55	310.06	0.00	0.60
1234	5	-291.27	310.83	0.78	0.40

Table S3. Details of the best fit models explaining the proportion of dead birds for the whole dataset. Variable numbers: 1 = Date, 2 = Moon, 3 = Rescue Patrol, 4 = Wind Speed, 5 = Holidays (note that it is not included in the best fit models)

Variables	df	logLik	AICc	Delta	Weight
13	3	-621.79	356.68	0	0.39
123	4	-619.25	357.32	0.65	0.28
134	4	-620.6	358.08	1.41	0.19
1234	5	-617.87	358.64	1.96	0.14

Table S4. Results of model averaging to explain the proportion of dead birds for the whole dataset.

Explanatory var.	Importance	Averaged estimate	S.E.	95% confidence intervals	
Intercept		-0.596	0.267	-1.123	-0.070
**Date**	1	0.061	0.009	0.043	0.079
**Rescue Patrol**	1	-2.310	0.073	-2.454	-2.167
Moon	0.42	-0.223	0.098	-0.417	-0.030
Wind Speed	0.34	0.006	0.003	-0.001	0.012
Holidays	0				

Table S5. Details of the best fit models assessing the effect of lighting management on the number of recued birds at the bridge of Phillip Island in 2009, 2012 and 2013. Variable numbers: 1 = Date, 2 = $(Date)^2$, 3 = Rescue Patrol, 4 = Lighting management

Variables	df	logLik	AICc	Delta	Weight
1234	6	-193.84	400.4	0	0.62
234	5	-195.43	401.37	0.97	0.38
